# TEST–RETEST RELIABILITY OF CAPABILITY MEASUREMENT IN THE UK GENERAL POPULATION

**DOI:** 10.1002/hec.3100

**Published:** 2014-09-09

**Authors:** Hareth Al-Janabi, Terry N Flynn, Tim J Peters, Stirling Bryan, Joanna Coast

**Affiliations:** a Health Economics Unit, School of Health and Population Sciences, University of BirminghamBirmingham, UK; b Centre for the Study of Choice, University of Technology SydneySydney, Australia; c School of Clinical Sciences, University of BristolBristol, UK; d School of Population and Public Health, University of British ColumbiaVancouver, Canada

**Keywords:** capability approach, outcomes, psychometrics, ICECAP, EQ-5D

## Abstract

Although philosophically attractive, it may be difficult, in practice, to measure individuals' capabilities (what they are *able* to do in their lives) as opposed to their functionings (what they actually do). To examine whether capability information could be reliably self-reported, we administered a measure of self-reported capability (the Investigating Choice Experiments Capability Measure for Adults, ICECAP-A) on two occasions, 2 weeks apart, alongside a self-reported health measure (the EuroQol Five Dimensional Questionnaire with 3 levels, EQ-5D-3L). We found that respondents were able to report capabilities with a moderate level of consistency, although somewhat less reliably than their health status. The more socially orientated nature of some of the capability questions may account for the difference. © 2014 The Authors Health Economics Published by John Wiley & Sons Ltd.

## 1. Introduction

The capability approach was pioneered in economics by Amartya Sen (Sen, 1987; Sen, 2009) to address limitations in the use of individual preferences to guide public policy (Sen, 1979). The capability approach advocates assessing the lives that people live in terms of their ‘functionings’ and ‘capabilities’. Functionings are the things that an individual ‘is’ or ‘does’. Capabilities represent an individual's freedom to carry out these functionings, whether or not the individual chooses to do so. Because capability (as opposed to functioning) encompasses the intrinsic value of freedom of choice, it is arguably superior to functioning alone as a basis for the assessment of well-being. Interest in using the capability approach in health economics has grown in recent years (Anand, 2005; Coast *et al*., 2008c). In particular, there has been progress in measuring capabilities for the purposes of resource allocation decisions (Coast *et al*., 2008a; Forder and Caiels, 2011).

An important challenge for any measure, whether it captures preferences, functioning or capability, is whether it can do this in a valid and reliable way. Various studies have investigated the validity of welfarist techniques, such as contingent valuation (Cookson, 2003; Smith, 2005) and discrete choice experiments (Bryan *et al*., 2000), and extra-welfarist techniques, such as preference-based measures of health status (Brazier *et al*., 1993; Brazier and Deverill, 1999). Measuring capability, however, presents an additional challenge. Capability measurement implies the quantification of something that is, strictly speaking, unobservable, at least directly (Hurley, 2001): the freedom or opportunities available to an individual. It requires an *ex ante* assessment, focusing on what an individual has the potential to do, rather than an assessment of their current functioning. Some recent studies report investigations of the construct validity of capability measures, showing that measured capability is higher in circumstances where one would expect it to be (for example, when incomes are higher, when health is better, etc.) (Coast *et al*., 2008b; Flynn *et al*., 2011; Al-Janabi *et al*., 2013b). However, to date, no study has examined whether capability can be reported reliably.

In this study, we investigated this issue of the reliability of capability responses by conducting a test–retest reliability study of a new capability measure (the Investigating Choice Experiments Capability Measure for Adults (ICECAP-A)) in a general population-based sample, using a widely used health status (functioning) measure, as a reference.

## 2. Data and Methods

Data for this study come from a web-based study conducted in March 2011 with individuals drawn from the UK general population. Respondents were recruited by Pure Profile (an online survey organisation) from their UK panel. Respondents self-completed the ICECAP-A capability measure (Al-Janabi *et al*., 2012) alongside the EuroQol Five Dimensional Questionnaire with three levels (EQ-5D-3L) health functioning measure (Brooks, 1996). The ICECAP-A measure comprises five questions covering an individual's ability to have stability, attachment, autonomy, achievement and enjoyment in their life. Individuals' response profiles can then be ‘scored’ between 0 (no capability) and 1 (full capability) using (UK) index values estimated previously (Flynn *et al*., 2013). The EQ-5D-3L comprises five questions on mobility, self-care, usual activities, pain/discomfort and anxiety/depression. Individuals' response profile can then be scored (on a scale where 0 is equivalent to death and 1 is equivalent to full health) using (UK) index values estimated previously (Dolan, 1997).

The measures were completed on two occasions, 2 weeks apart. A 2-week interval is thought to be long enough, so that respondents do not simply remember their previous response, but short enough that there will be little real change (Streiner and Norman, 2003). Thus, for a reliable assessment of capability or health, one would expect near-identical answers at baseline and follow-up for those individuals who experienced no real change in capability or health. Respondents also provided socio-demographic information and a self-assessment of whether their quality of life or health had changed over the 2 weeks. Specifically, they were asked:

- Has your well-being or quality of life changed over the last 2 weeks? (yes/no)
- Has your health changed over the last 2 weeks? (yes/no)

The questions allowed those who reported a real change in their life to be removed from the study sample for this analysis. The study protocol was approved by the University of Birmingham's Life and Health Sciences Ethical Review Committee (ERN_08-93).

A reliable (or consistent) response to a question was defined as one where the same response level was provided at both time periods. Test–retest reliability of responses to individual capability questions was estimated by calculating chance-corrected agreement between the two sets of responses to the capability questions. Linear weighted kappa statistics were used to account for the fact that inconsistent responses could vary in their level of inconsistency (for example, a level 4 response and a level 2 response are both inconsistent with a level 1 initial response, but the former is more inconsistent). The same approach was used for the health status questions. Quadratic weighted kappa statistics were calculated for both measures as a sensitivity analysis. The reliability of the response to the measure as a whole was calculated by estimating the intra-class correlation (ICC) between respondents' baseline and 2-week capability index scores.

Regression analysis was used to investigate whether unreliable responses could be explained by socio-demographic characteristics of respondents. Two ordered categorical variables were created (one for the ICECAP-A and one for the EQ-5D-3L). These variables recorded the number of attributes (ranging from 0 to 5) in which an inconsistent response was provided for each individual. Two ordinal logistic regressions (one for the ICECAP-A and one for the EQ-5D-3L) were then used to study whether the resulting variables were associated with individuals' age, sex and education. All analyses were conducted in Stata v10.

## 3. Results

**Figure 1:**
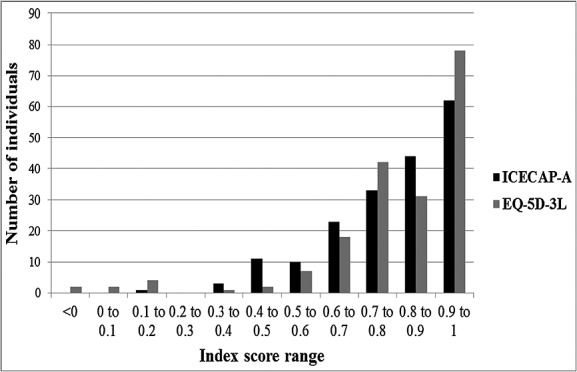
The distribution of ICECAP-A index scores and EQ-5D-3L index scores in the sample at baseline (*n* = 187)

Two hundred and eighty-six individuals were approached for the survey in January 2011, and of these, 237 (83%) completed the baseline and follow-up questions. The completing sample was broadly representative of the UK population in terms of sex (52% male subjects) and age (23% over 65 years). At baseline, the sample reported a mean ICECAP-A index score of 0.78 and mean EQ-5D-3L score of 0.80. At the 2-week follow-up, 50 (21%) individuals reported that their health, quality of life or well-being had changed in the past 2 weeks. These individuals were excluded from the reliability analysis. Figure1 shows the distribution of index scores on the ICECAP-A and EQ-5D-3L for the respondents at baseline.

The data show that many more inconsistent responses occurred with the ICECAP-A measure, relative to the EQ-5D-3L (Table I). Indeed, most (84%) respondents provided an inconsistent response to at least one of the ICECAP-A questions. On the EQ-5D-3L, a minority (38%) provided an inconsistent response to at least one of the questions. These data, however, mask the much greater chance of reporting inconsistent capability data. This arises because of the greater variability in baseline response to the capability questions. At baseline, 82 different capability states were reported compared with 25 different health states. In part, this arose because individuals were less likely to be bunched at the ceiling (top state) of the ICECAP-A. For example, five respondents (3%) selected the top ICECAP-A state at both time points, while 66 respondents (35%) selected the top EQ-5D-3L state at both time points. Furthermore, on the ICECAP-A measure, respondents could select one of four response categories (as opposed to three on the EQ-5D-3L).

**Table I:** Frequency of inconsistent responses

	Number of inconsistent responses per measure					
	0	1	2	3	4	5
ICECAP-A	29	61	59	25	9	4
EQ-5D-3L	116	50	15	4	2	0

Table II shows the level of agreement on the attributes for the 187 respondents who completed the ICECAP-A and EQ-5D-3L and reported no changes in well-being or health over the period. The reliability of the capability questions, which takes into account higher level of inherent variability, is in the range of 0.52 (autonomy) to 0.61 (stability). The reliability of the health status questions is somewhat higher, in the range of 0.60 (usual activities) to 0.79 (mobility). Using quadratic weights for the kappa statistics resulted in higher reliability coefficients for the ICECAP-A items (0.63–0.72) and little change for the EQ-5D-3L items (0.62 to 0.79). The ICC for the measures (index scores) as a whole was 0.72 for the ICECAP-A and 0.83 for the EQ-5D-3L.

**Table II:** Reliability of the ICECAP-A and EQ-5D-3L items

	Agreement (%)	Weighted kappa (k)
ICECAP-A		
Stability	89.8	0.61
Attachment	88.8	0.57
Autonomy	87.8	0.52
Achievement	88.1	0.53
Enjoyment	88.1	0.54
EQ-5D-3L		
Mobility	92.5	0.79
Self-care	97.5	0.77
Usual activities	93.0	0.60
Pain/Discomfort	93.5	0.76
Anxiety/Depression	91.7	0.67

Table III shows the results of the regressions of the number of inconsistent responses on socio-demographic characteristics of respondents. There is no strong evidence of associations between age, sex or education and the propensity for someone to provide a consistent capability response, and only age is associated with the likelihood of providing a consistent health status response. In this case, older people were more likely to provide inconsistent responses to health questions than younger people.

**Table III:** Regression estimates of the associations between reliability (inconsistent responses) and socio-demographic characteristics

	Age	Sex	Education
Model 1: inconsistent responses on ICECAP-A	0.019 (0.604)	0.366 (0.155)	−0.264 (0.080)
Model 2: inconsistent responses on EQ-5D-3L	0.130 (0.003)	0.032 (0.912)	−0.318 (0.058)

Note: Cell values represent the coefficients from the univariable ordinal regressions with *p*-values in parentheses.

## 4. Discussion

As new approaches to conceptualising health and well-being start to be used in health economics, their measurement properties should be established. In this study, we investigated the test–retest reliability of capability measurement for the general adult population. We found that the reliability of a simple measure of adult capability (the ICECAP-A) was slightly lower than that for a commonly used health functioning measure (the EQ-5D-3L) but not obviously affected by age, sex or education.

Although the reliability of responses to the ICECAP-A attributes was somewhat lower compared with the EQ-5D-3L, agreement was still ‘moderate’ to ‘substantial’ (Landis and Koch, 1977). In terms of the scale as a whole, opinions differ as to what constitutes an acceptable reliability coefficient. Studies investigating the reliability of clinical scales have found reliability coefficients in the range of 0.7 and 0.8 (Brazier *et al*., 1992; Hurst *et al*., 1997; Hughes, 2007), generally in the range observed for the ICECAP-A and EQ-5D-3L. If measures are intended to be used in research (as opposed to informing a specific clinical decision), then lower coefficients can be tolerated because conclusions will typically be based on mean scores of many individuals and on multiple studies, both of which serve to reduce measurement variability (Streiner and Norman, 2003). Further, the reliability estimates in this study may be artificially low if we failed to screen out individuals who experienced real change in their capability. This would have happened if an individual's real capability changed but that individual did not self-report change in well-being, quality of life or health.

The more socially orientated nature of the ICECAP-A questions compared with the EQ-5D-3L questions could explain the lower reliability of the ICECAP-A. Scales that tap social constructs have been found to have lower levels of reliability than those that tap physical constructs (Brazier *et al*., 1992). This could be because of social traits (such as one's perception of autonomy) being inherently less stable than physical traits (such as mobility). Evidence suggests that measures of stable traits such as intelligence quotient or extraversion display much higher reliabilities than measures of mild pathological states such as anxiety (Streiner and Norman, 2003). This suggests that developing highly reliable measures of social (or combined) capability may be challenging. However, it also suggests that measures of physical capabilities (or internal capabilities (Alkire, 2002)) might be expected to be more reliable than measures of social capabilities.

The regression results showed that socio-demographic characteristics were, in general, not associated with how many unreliable responses an individual provided. With the exception of an inference that advancing age may be associated with less reliable responses to the EQ-5D-3L, the regressions shed little light on the reasons behind inconsistent responses. This may, however, be because of the sample size employed in this study.

The nature of the sample needs to be considered when interpreting the results. Given that this was a small study, comparisons with the wider population are likely to exhibit some unrepresentativeness on at least one criterion. Furthermore, web-based administration of the measures is not always used in practice (currently). However, we have no reason to suspect that reliability would differ between web-based and paper-based administrations.

Qualitative work to understand the response to capability questions suggests that individuals have varying interpretations of the capability concept (Al-Janabi *et al*., 2013a). Individuals vary in terms of the time frame applied when thinking about their capability and what constraints are relevant in identifying their capability set. An inconsistent interpretation of capabilities across respondents that is not attributable to systematic differences by, say, age and education is therefore consistent with these findings. Investigating this issue and comparing the psychometric performance of physical and social capabilities offer potential areas of further investigation.

## 5. Conclusion

Capability status was reported slightly less reliably than health (functioning) status. In part, this may be because of the more socially (as opposed to physically) orientated nature of the capability (ICECAP-A) measure. Nevertheless, a wider range of capability states was observed in this sample relative to the number of health states suggesting that the capability measure may have had greater sensitivity to subtle differences in quality of life in this sample. For both measures, the reliability coefficients were within generally acceptable bounds for research studies.
